# Vascular Mesenchymal Stromal Cells and Cellular Senescence: A Two-Case Study Investigating the Correlation Between an Inflammatory Microenvironment and Abdominal Aortic Aneurysm Development

**DOI:** 10.3390/ijms252312495

**Published:** 2024-11-21

**Authors:** Gabriella Teti, Riccardo Camiletti, Valentina Gatta, Aurora Longhin, Mirella Falconi

**Affiliations:** 1 Department of Biomedical and Neuromotor Sciences (DIBINEM), University of Bologna, 40126 Bologna, Italy; riccardo.camiletti@studio.unibo.it (R.C.); valentina.gatta6@unibo.it (V.G.); aurora.longhin2@unibo.it (A.L.); 2 Department of Medical and Surgical Sciences (DIMEC), University of Bologna, 40126 Bologna, Italy; mirella.falconi@unibo.it

**Keywords:** abdominal aortic aneurysm, cellular senescence, vascular inflammation remodeling, mesenchymal stromal cells

## Abstract

An abdominal aortic aneurysm (AAA) is described as a gradual and localized permanent expansion of the aorta resulting from the weakening of the vascular wall. The key aspects of AAA’s progression are high proteolysis of the structural elements of the vascular wall, the depletion of vascular smooth muscle cells (VSMCs), and a chronic immunoinflammatory response. The pathological mechanisms underpinning the development of an AAA are complex and still unknown. At present, there are no successful drug treatments available that can slow the progression of an AAA or prevent the rupture of the aneurysmal vascular wall. Recently, it has been suggested that endothelial cellular senescence may be involved in vascular aging and vascular aging diseases, but there is no clear correlation between cellular senescence and AAAs. Therefore, the aim of this study was to identify the presence of senescent cells on the vascular wall of aneurysmatic abdominal aortas and to correlate their distribution with the morphological markers of AAAs. Pathological and healthy segments of abdominal aortas were collected during repair surgery and immediately processed for histological and immunohistochemical analyses. Hematoxylin/eosin, Verhoeff–van Gieson, and Goldner’s Masson trichrome staining procedures were carried out to investigate the morphological features related to the pathology. Immunohistochemical investigations for the p21^cip1/waf1^, p53, and NFkB markers were carried out to selectively identify positive cells in the vascular wall of the AAA samples related to cellular senescence and an inflammatory microenvironment. The results revealed the presence of a few senescent vascular cells on the aneurysmatic wall of the abdominal aortas, surrounded by a highly inflamed microenvironment that was highly expressed in the tunica media and adventitia of both pathological and healthy segments. Our data demonstrate the presence of senescent vascular cells in AAA samples, which could enhance the promotion of a high inflammatory vascular microenvironment, supporting the evolution of the pathology. Although this study was based on only two cases, the results highlight the importance of targeting cellular senescence to reduce an inflammatory microenvironment, which can support the progression of age-related diseases.

## 1. Introduction

An abdominal aortic aneurysm (AAA) is a vascular pathology that is described as a gradual and permanent expansion of the aorta, resulting from the weakening of the vascular wall [1,2]. Extracellular matrix (ECM) breakdown, inflammation, the switching of the vascular smooth muscle cell (SMC) phenotype, oxidative stress, and neovascularization are key aspects in the progression of the pathology [1,2,3]. It is thought that these biological mechanisms trigger the degradation of elastic fibers and alterations in the collagen composition in the three vascular layers (tunica intima, media, and adventitia), reducing the flexibility of the aortic wall [3,4]. Currently, there are no effective drugs that can delay or prevent the development of an AAA, and, in the most severe cases, surgical or endovascular repair is the only treatment option available.

The repair process following a vascular injury has been associated with various cardiovascular diseases [5]. The vascular stem cells (VSCs) residing in blood vessels are crucial for vascular remodeling. They include endothelial progenitor cells (EPCs), smooth muscle progenitor cells (SMPCs), vascular mesenchymal stromal cells (MSCs), and pericytes. In the case of vascular damage, MSCs can differentiate into several types of vascular cells and form neointima during vascular repair [6]. Due to their self-renewal and differentiation abilities, MSCs participate in maintaining tissue homeostasis and contribute to tissue regeneration in response to injury [7,8]. Under physiological conditions, vascular MSCs are mainly localized within the adventitial layer and the medial layer, while during vascular remodeling under pathological conditions, they migrate and differentiate toward SMCs, aiding in neointima formation [7,9,10].

Cellular senescence is described as a permanent growth arrest in the cell cycle, although cells keep their viable and metabolically active state [11,12]. It is defined by several phenotypic changes, such as an enlarged cell size, elevated senescence-associated beta-galactosidase (SA-β-gal) activity, the formation of telomere-associated foci, and an increased expression of the cyclin-dependent kinase inhibitors (CDKi) p16^INK4^ and p21^cip1/waf1^ [13]. Various stress conditions that induce DNA damage [14], including the production of reactive oxygen species (ROS) [15], oncogene activation [16], telomere attrition [17], and metabolic dysregulation [12], could be responsible for triggering premature senescence. The accumulation of senescent cells contributes to the secretion of pro-inflammatory cytokines, chemokines, growth modulators, and matrix metalloproteinases, representing the senescence-associated phenotype (SASP) [18,19]. The SASP severely changes the local microenvironment, promoting aging and the progression of age-related diseases.

Recently, it has been proposed that cellular senescence could be involved in the onset and progression of some cardiovascular diseases such as atherosclerosis [20,21], endothelial dysfunction [19], and vascular aging and calcification [22]. Advanced atherosclerosis is characterized by the presence of senescent endothelial cells (ECs), VSMCs, foam cells, and macrophages, fostering a pro-inflammatory environment that activates matrix metalloproteinases (MMPs) together with macrophages. This leads to conditions that strongly increase susceptibility to plaque [20].

Previous studies have demonstrated a senescent phenotype in VSMCs and endothelial cells isolated from AAA explants and cultured in vitro [23]. Recently, it has been found that vascular MSCs isolated from AAA explants show a higher expression of the CDKi p21^cip1/waf1^, greater ROS production, an enlarged size, and reduced autophagy, in agreement with a senescent phenotype [24]. Although some in vitro evidence correlates AAAs and cellular senescence, clear scientific evidence in ex vivo samples is still lacking. A full understanding of the molecular mechanisms promoting cellular senescence and its correlation with the pathophysiology of AAAs will allow the design of new drugs that minimize the harmful consequences of senescence, bringing benefits to patients.

With the aim of closely connecting AAAs and cellular senescence, we investigated the presence of senescent cells on the vascular wall of aneurysmatic abdominal aortas and correlated their distribution with the morphological markers of AAAs. To our knowledge, this study represents one of the few attempts to evaluate senescent cells in the aneurysmatic vascular wall, providing information on the real in vivo situation of the pathological vascular wall. Histological and immunohistochemical analyses were carried out on paraffin-embedded sections of AAAs and healthy vascular samples to demonstrate the morphological features and the presence of senescent and inflammatory markers related to the development of AAAs.

## 2. Results

### 2.1. Hematoxylin and Eosin (HE) Morphological Evaluation

**Figure 1:**
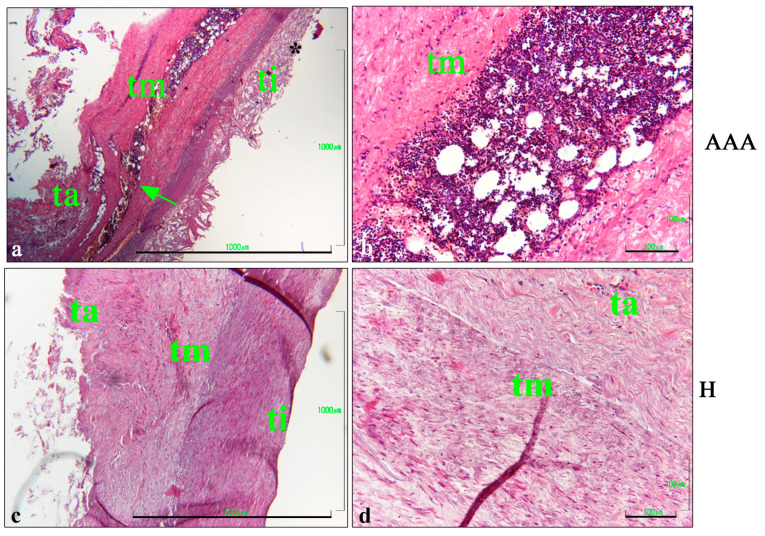
HE staining of aortic vascular tissues. (**a**) Representative images of AAA sections after HE staining, showing the tunica intima, which was characterized by the presence of cholesterol crystals (*); and the tunica media, with a large cluster of inflammatory cells (arrow). The tunica adventitia was partially preserved (bar: 1000 μm). (**b**) Details of the AAA’s tunica media, showing inflammatory cells (bar: 100 μm). (**c**) Representative images of H abdominal aorta sections after HE staining, showing a thick layer of the tunica intima and media (bar: 1000 μm). (**d**) Details of the tunica media, showing the integrity of the layer and the lack of inflammatory infiltration (bar: 100 μm). ti, tunica intima; tm, tunica media; ta, tunica adventitia.

A light microscopy analysis of HE-stained vascular tissues showed a highly damaged morphology in AAA samples, underlining the lack of integration between the tunica intima, media, and adventitia (Figure 1a,b). Extensive inflammatory infiltrate was detected in the tunica media (Figure 1a,b). On the contrary, the healthy samples (H) showed a better integration of the three vascular layers and the lack of inflammatory cells in the vascular wall (Figure 1c,d).

### 2.2. Verhoeff–Van Gieson Morphological Evaluation 

**Figure 2:**
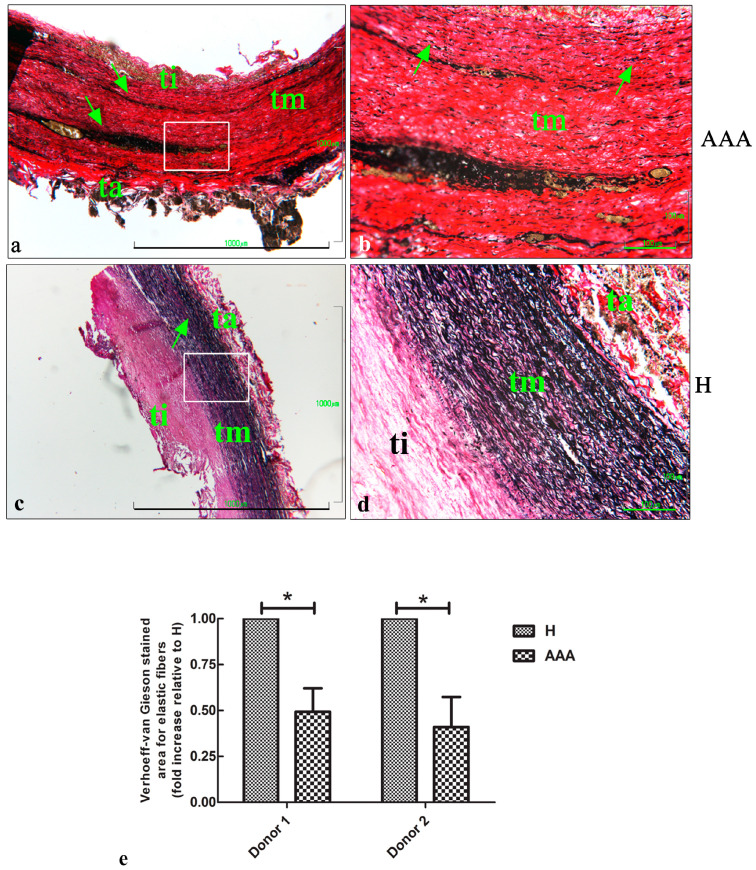
Verhoeff–van Gieson staining of aortic vascular tissues. (**a**) Representative images of AAA sections after Verhoeff–van Gieson staining, showing a great reduction in the number of elastic fibers in the tunica intima and the high infiltration level of inflammatory cells (arrows) (bar: 1000 μm). (**b**) Details of the tunica media, showing the presence of thin and short elastic fibers (arrows) and inflammatory infiltration (bar: 100 μm). (**c**) Representative images of healthy (H) sections after Verhoeff–van Gieson staining, showing a thick layer of elastic fibers (black lines) in the tunica media (arrow) (bar: 1000 μm). (**d**) Details of the elastic fibers in the tunica media of H samples (bar: 100 μm). (**e**) Quantitative analysis of the elastic areas in the AAA and H samples of the two donors involved in the study. The data are represented as the fold increases compared with the control samples (* *p* < 0.05). ti, tunica intima; tm, tunica media; ta, tunica adventitia.

In order to investigate the presence of elastic fibers in the vascular wall, the Verhoeff–van Gieson staining procedure was carried out. The results showed a great reduction in the number of elastic fibers in the tunica media of the AAA samples (Figure 2a,b) compared with the H samples, for which several elastic fibers were detected (Figure 2c,d) in the tunica media. The AAA samples still demonstrated a high number of inflammatory cells localized in the tunica media (Figure 2a,b). A quantitative analysis of the area characterized by the presence of elastic fibers showed a 0.5-fold reduction in the AAA samples compared with the H samples (Figure 2e) for both the donors involved in the study.

### 2.3. Goldner’s Masson Morphological Evaluation 

**Figure 3:**
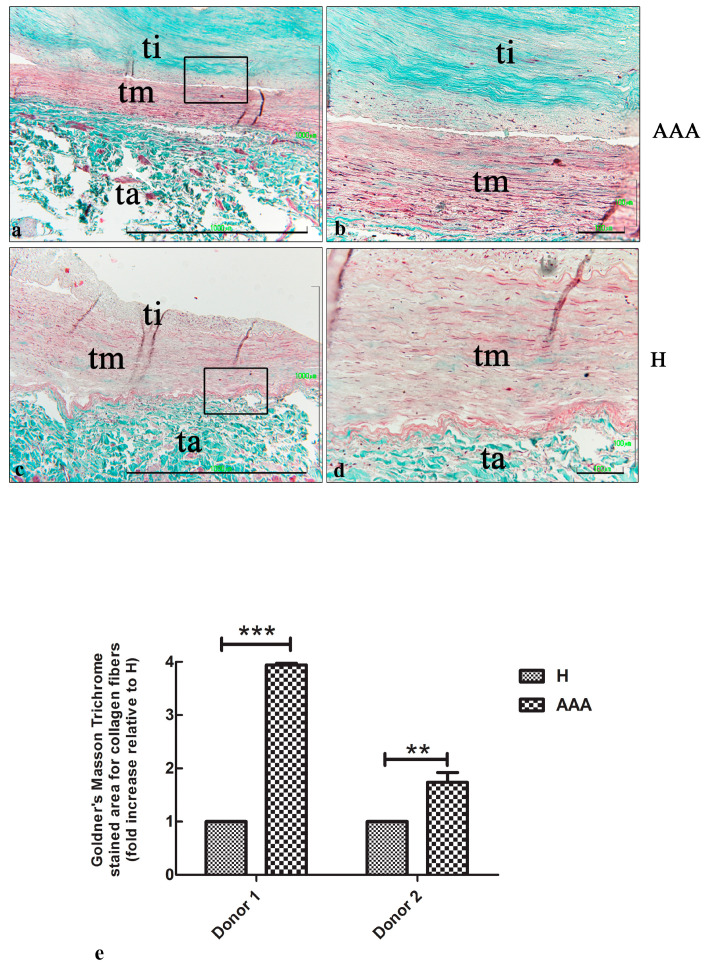
Goldner’s Masson staining of aortic vascular tissues. (**a**) Representative images of AAA sections after Goldner’s Masson staining, showing a large fibrotic area in the tunica intima, which was characterized by the deposition of collagen fibers (colored in green). Note the thin layer of tunica media (bar: 1000 μm). (**b**) Details of the tunica intima, in which a parallel oriented deposition of collagen fibers was detected (green color) (bar: 100 μm). (**c**) Representative images of H sections after Goldner’s Masson staining, showing a very low number of collagen fibers (green) in the tunica intima and media (bar: 1000 μm). (**d**) Details ofthe vascular wall in which the green color corresponds to collagen deposition that was slightly observed in the tunica media (100 μm). (**e**) Quantitative analysis of the collagenic areas in the AAA and H samples of the two donors involved in the study. The data are represented as the fold increase compared with the control samples (*** *p* < 0.001; ** *p* < 0.01). ti, tunica intima; tm, tunica media; ta, tunica adventitia.

To evaluate the presence of a fibrotic area in the vascular wall of AAAs and healthy abdominal aorta samples, Goldner’s Masson trichrome staining was carried out. Figure 3 shows an extensive area of collagen fibers in the tunica intima of AAA samples (Figure 3a,b). On the contrary, just a few lines of collagen fibers were detected in H samples (Figure 3c,d). A quantitative analysis showed a two- to fourfold increase in the fibrotic areas in the AAA samples compared with the H samples (Figure 3e).

### 2.4. Ultrastructural Analysis Using TEM

**Figure 4:**
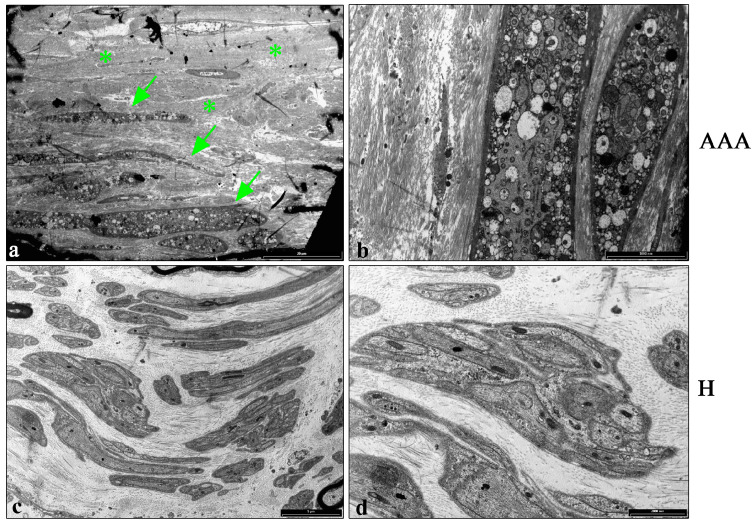
TEM analysis of aortic vascular tissues. (**a**) Representative TEM images of the tunica media of AAA samples, demonstrating the presence of necrotic cells in the tunica media of the vascular walls (arrows). The cells are surrounded by a large and discontinuous area of collagen fibers (*) (bar: 20 μm). (**b**) Details of the necrotic cells in the tunica media in the vascular wall of AAA specimens. Several vacuoles of different densities and sizes were detected in necrotic cells (bar: 5000 nm). (**c**) Representative TEM images of the tunica media in the vascular wall of H samples. Clusters of smooth muscles cells surrounded by a thin layer of collagen fibers were detected (bar: 5 μm). (**d**) Details of the smooth muscle cells, in which contractile fibers inside the cells were detected (bar: 2000 nm).

A TEM analysis was carried out to visualize the ultrastructural changes in the vascular wall of AAAs compared with the healthy samples. Figure 4 shows the presence of necrotic smooth muscle cells in the tunica media of AAAs (Figure 4a,b), characterized by the presence of several vacuoles of different sizes and densities. The cells were surrounded by a large and discontinuous area of collagen fibers (Figure 4a,b).

H samples showed the presence of clusters of smooth muscle cells in the tunica media, in which the contractile filaments were detected in the cytoplasm (Figure 4c,d). The cells were surrounded by a thin layer of regularly oriented collagen fibers (Figure 4c,d), in agreement with the Goldner’s Masson staining results.

### 2.5. Immunohistochemical Expression of p21^cip1/waf1^ CDKi Protein in the AAAs’ Vascular Wall

**Figure 5:**
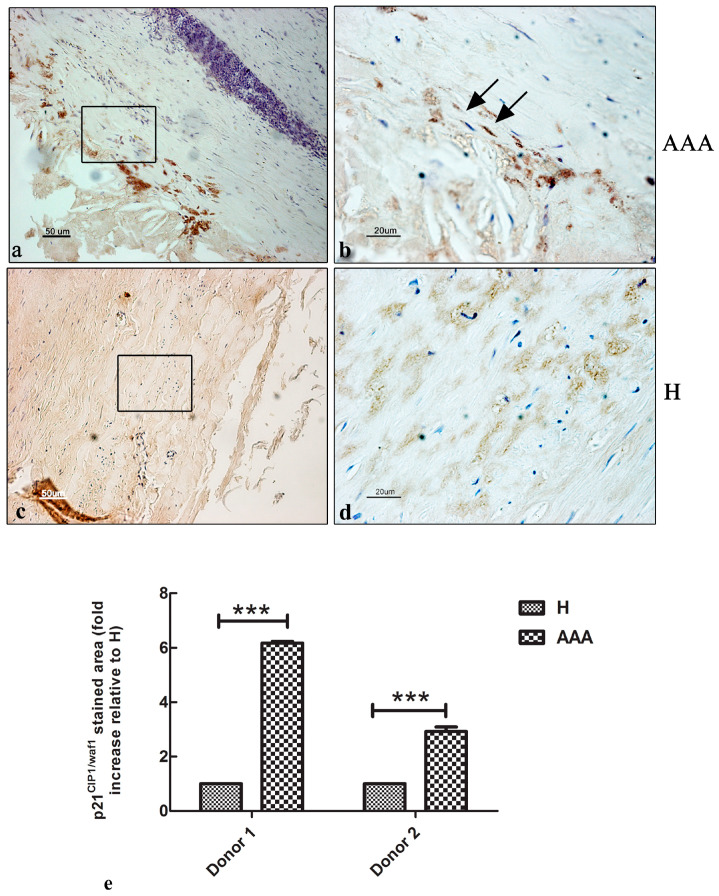
The expression of the p21^cip1/waf1^ senescent marker in aortic vascular tissues. (**a**) Representative images of the immunohistochemical expression of the p21^cip1/waf1^ marker in the vascular wall of AAA samples. A few positive cells were detected in the tunica intima (black arrows), media, and adventitia (bar: 50 μm). (**b**) Details of the tunica intima, showing the presence of a few p21^cip1/waf1^-positive nuclei (bar: 20 μm). (**c**) Representative images of the immunohistochemical expression of the p21^cip1/waf1^ marker in the vascular wall of H samples. No signal was detected in any of the three layers of the vascular wall (bar: 50 μm). (**d**) Details of the tunica media and adventitia, showing the lack of positive nuclei (bar: 20 μm). (**e**) Quantitative analysis of the p21^cip1/waf1^ stained areas in the AAA and H samples of the two donors involved in the study. The data are represented as the fold increase compared with the control samples (*** *p* < 0.001). All the slides were counterstained with hematoxylin to detect nuclei (blue color). Ti, tunica intima; tm, tunica media; ta, tunica adventitia.

To evaluate the expression and the localization of the senescent marker p21^cip1/waf1^ in the vascular wall of the AAA samples, an immunohistochemical analysis was carried out. Different from what was expected, a low number of positive cells was detected at the boundary of the tunica intima and media, and in the tunica adventitia (Figure 5a,b). The control samples showed no positive cells in the tunica intima or media (Figure 5c,d). A quantitative analysis of the area stained with the p21^cip1/waf1^ protein showed an increase in the signal of up to sixfold compared with the H segments, although there was a difference between the two donors involved in the study (Figure 5e).

### 2.6. Immunohistochemical Expression of p53 Protein in AAAs’ Vascular Wall

**Figure 6:**
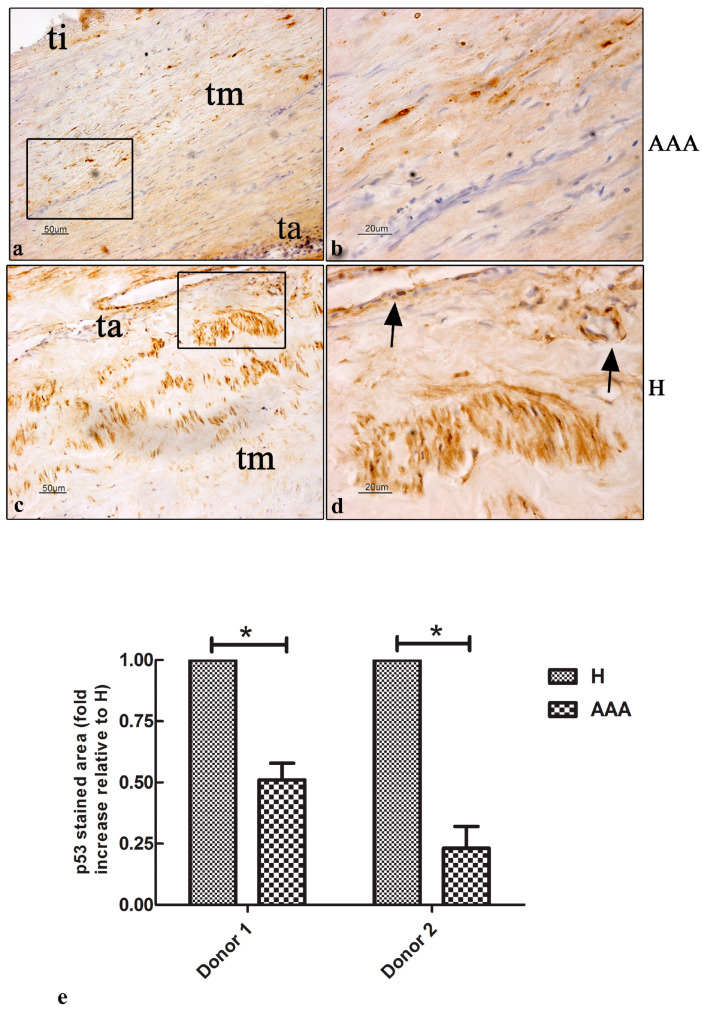
The expression of the p53 protein in aortic vascular tissues. (**a**) Representative images of the immunohistochemical expression of the p53 marker in the vascular wall of AAA samples. A few positive nuclei were detected in the three layers of the vascular wall (bar: 50 μm). (**b**) Details of the tunica media, showing positive nuclei (bar: 20 μm). (**c**) Representative images of the immunohistochemical expression of the p53 marker in the vascular wall of H samples. A high level of expression was detected in the nuclei of the tunica media and adventitia (bar: 50 μm). (**d**) The vasa vasorum of the tunica adventitia showed a positive p53 signal (black arrows) (bar: 20 μm). (**e**) Quantitative analysis of the p53 stained areas in the AAA and H samples of the two donors involved in the study. The data are represented as the fold increase compared with the control samples (* *p* < 0.05). All the slides were counterstained with hematoxylin to detect nuclei (blue color). Ti, tunica intima; tm, tunica media; ta, tunica adventitia.

Due to the key role of the p53 protein in the DNA damage response (DDR) and the correlation with p21^cip1/waf1^ expression, we decided to investigate the presence and localization of the p53 protein in the vascular wall of AAA samples using immunohistochemistry. The AAA samples showed a weak signal in all three layers of the vascular wall (Figure 6a,b). A few positive nuclei were detected in the tunica media (Figure 6b). Surprisingly, a high signal of p53 expression was detected in the tunica media and adventitia of H samples (Figure 6c,d). The vasa vasorum of the tunica adventitia showed a positive signal (Figure 6c,d). A quantitative analysis demonstrated a 0.5-fold reduction in the p53 expression in AAA samples compared with H samples (Figure 6e).

### 2.7. Immunohistochemical Expression of NFkB Factor in AAAs’ Vascular Wall

**Figure 7:**
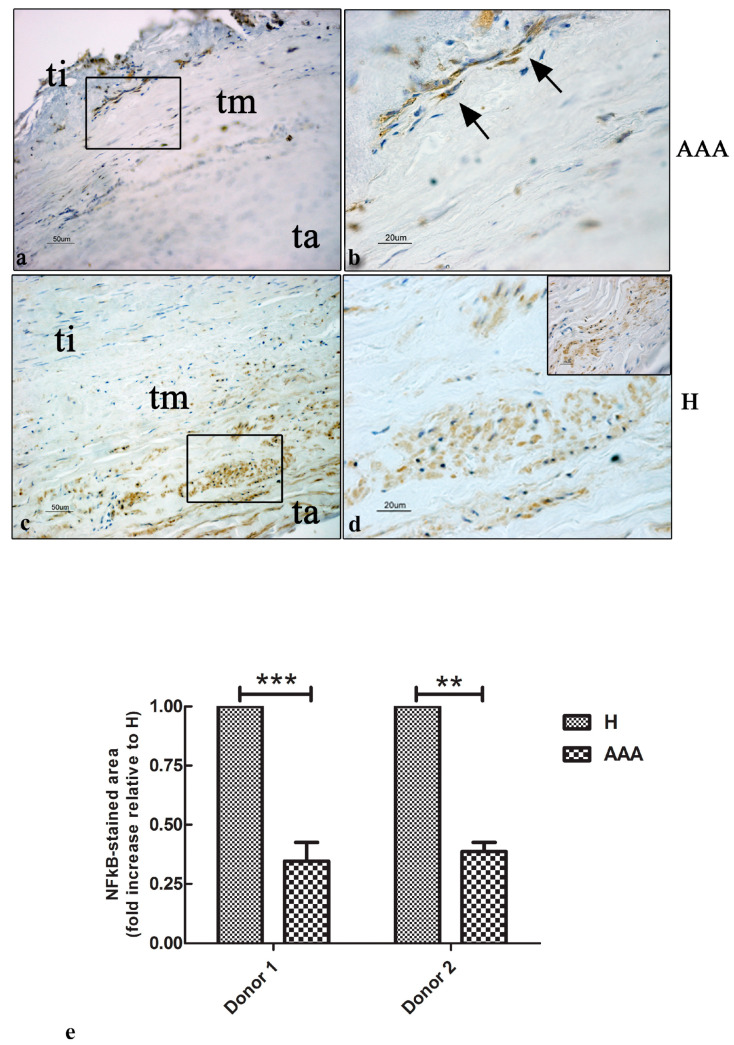
The expression of the NFkB marker in aortic vascular tissues. (**a**) Representative images of the immunohistochemical expression of the NFkB marker in the vascular wall of AAA samples. A few positive nuclei were detected and were mainly localized between the tunica intima and the tunica media (bar: 50 μm). (**b**) Details of the tunica intima, showing a few positive nuclei (black arrows) (bar: 20 μm). (**c**) Representative images of the immunohistochemical expression of the NFkB marker in the vascular wall of H samples. A high level of expression was detected in the nuclei of the tunica media and adventitia (bar: 50 μm). (**d**) Details of the tunica media, showing cells expressing the NFkB factor (bar: 20 μm). Some positive cells of the tunica adventitia were also detected (square; bar: 20 μm). (**e**) Quantitative analysis of the p53 stained areas in the AAA and H samples of the two donors involved in the study. The data are represented as the fold increase compared with the control samples (*** *p* < 0.001; ** *p* < 0.01). All the slides were counterstained with hematoxylin to detect nuclei (blue color). Ti, tunica intima; tm, tunica media; ta, tunica adventitia.

NFkB is one of the first inflammatory factors to be stimulated following damage. We decided to investigate the presence and localization of NFkB-positive cells in the vascular wall of healthy and pathological abdominal aortas using immunohistochemistry. The results showed a few positive cells in the AAA samples, mainly localized at the boundary between the tunica intima and the tunica media of the pathological vascular wall (Figure 7a,b). The healthy abdominal aorta samples showed a high number of positive cells, mainly localized in the tunica media and tunica adventitia (Figure 7c,d). A quantitative analysis demonstrated a 0.5-fold reduction in the NFkB expression of the AAAs’ vascular wall compared with the H samples (Figure 7e).

## 3. Discussion

The aim of this study was to demonstrate the presence of cellular senescence in the vascular wall of AAAs and to detect which cellular populations mainly show the senescent phenotype. Although some scientific studies have already reported the presence of cellular senescence in endothelial cells [19], smooth muscle cells [23], and MSCs [24], they have been mainly based on in vitro experiments and in vivo mouse models [25], while studies based on an analysis of human biopsies are missing. Our study mainly focused on the investigation of the morphological features of AAAs and the senescent phenotype using human AAA biopsies, with the final goal of demonstrating a correlation between AAA development and cellular senescence. The hypothesis was that the persistence of senescent cells in the arterial vascular wall during aging through the secretion of SASP factors could trigger low, silent, and chronic inflammation, which would promote tissue dysfunction and affect vascular remodeling.

The molecular mechanisms of AAA development are still mainly unknown. Proteolysis, oxidative stress, inflammatory immune responses, and VSMCs’ death are the main morphological hallmarks related to aneurysms’ onset and development [1]. Our samples clearly demonstrated the morphological characteristics of AAAs. The HE data showed the presence of a large inflammatory infiltrate in the arterial vascular wall and the lack of integration between the tunica intima, media, and adventitia in the biopsies of AAAs, in agreement with the scientific literature [26]. Chronic inflammation caused by the infiltration and activation of various immune cells is an important driver of AAAs, although the mechanisms of their activation and function are still far from being understood [26]. Recently, the pivotal role of perivascular adipose tissue (PVAT) in the onset of leukocyte infiltration has been suggested. As a consequence of vascular damage, the expression of inflammatory factors such as resistin, leptin, cytokines, and chemokines (14) is triggered by PVAT, which enhances the infiltration of inflammatory cells, including neutrophils, macrophages, natural killer cells (NK cells), dendritic cells (DCs), T and B lymphocytes, and mast cells [26,27. Furthermore, the composition and activation status of immune cells infiltrating the aortic wall during AAA development is dynamic and changes throughout the course of disease development [26]. The inflammatory infiltrate is responsible for the secretion of pro-inflammatory mediators such as IL-1β, IL-6, TNF, IL-12, IL-23, MMPs, NOS2, and chemokines, including CCL2 and CXCL1, in turn regulating the recruitment and activation of other immune cells [27]. Neutrophil elastases are mainly responsible for the elastic degradation of the aneurysmatic vascular wall [28]. To verify the lack of abundance of elastic components in our samples, Verhoeff–van Gieson staining was carried out. The results demonstrated a strong reduction in the elastic fiber area in the tunica media of the AAA samples, in agreement with the intense proteolysis that characterizes the vascular wall of AAAs [28]. Moreover, AAAs are characterized by the degradation of connective tissue, mainly of the elastin fibers, through the activation of various proteases such as plasmin, elastase, cathepsins, and matrix MMPs, along with a reduction in the expression of elastogenic proteins [29,30,31,32]. Modifications in Collagen XII, thrombospondin 2, aortic carboxypeptidase-like protein, periostin, fibronectin, and tenascin extracellular matrix components have been recently demonstrated by a proteomic study in human AAA specimens [30]. As a compensatory reaction to the inflammatory environment, the aortic wall undergoes adverse vascular remodeling, which consists of a deep reduction in the vascular tunica media, SMC loss, elastin fragmentation, conversion of fibroblasts to myofibroblasts, an increase in the production of reactive oxygen species (ROS), and an increase in collagen deposition [33]. Adverse vascular remodeling is considered to be a pivotal factor during the onset and development of AAAs. In agreement with the scientific literature, Goldner’s Masson trichrome staining results demonstrated higher collagen deposition in the AAA samples compared with the healthy vascular segments, while the TEM analysis showed several necrotic cells in the tunica media of AAA samples, corresponding to dead VSMCs [33,34]. The loss of VSMCs is one of the major features of AAAs’ development and progression, and it is attributed to increased cell death driven by apoptosis and necroptosis [34]. In an ANG-II induced mouse AAA model, it has been demonstrated that the extrinsic apoptotic pathway, generally activated by cytotoxic drugs, UV radiation, and the intrinsic apoptotic pathway, and generally triggered by DNA damage, oncogene activation and hypoxia, are upregulated during the early events of AAAs’ onset [30]. Furthermore, a tight connection between endoplasmic reticulum stress and apoptosis of VSMCs in AAA progression has been suggested [30].

Recently, a role of VSMC-accelerated senescence has been suggested in inducing cell death [23,35]. In vitro experiments have already demonstrated a senescent phenotype in MSCs isolated from human AAA samples, with a high level of ROS production and impaired autophagy [24]. Senescent MSCs in the vascular wall of AAAs could strongly contribute to the progression of the pathology, mainly through the secretion of SASP factors. To demonstrate the presence of senescent cells in the vascular walls of human AAA samples, immunohistochemical investigations for the p21^cip1/waf1^ senescent marker were carried out. Surprisingly, the results showed a low signal of positive p21^cip1/waf1^ cells in the vascular wall of human AAA segments, mainly localized between the tunica intima and the tunica media, while almost no positive cells were detected in healthy abdominal aorta samples. Despite the quantitative analysis demonstrating a sixfold increase in the p21^cip1/waf1^ expression in pathological samples compared with control ones, we found a large difference in the results between the two donors involved in this study. Although we expected a low positive signal, which was in line with the reduced number of cells in the vascular wall that could proliferate or enter into a senescent state, the real number observed was much too low. As already demonstrated by our TEM analysis, the pathological vascular walls were characterized by high cell death; therefore, we suggest that the low level of p21^cip1/waf1^ expression is correlated with the low number of cells still present in the vascular wall.

The expression of the senescent marker p21^cip1/waf1^ is strictly linked to the DDR, is induced by DNA damage, and is under the control of the transcription factor p53, which regulates the stop of the cell cycle in the G1 or G2 phase and promotes cytokine secretion, producing SASPs [36]. Therefore, we investigated the expression and localization of the p53 protein in AAA and healthy vascular samples. The results showed a few positive cells in the tunica media and a high positive signal in the tunica adventitia and the vasa vasorum, suggesting a potential role of damage to the vasa vasorum in the development of the pathology.

Senescence and inflammation phenotypes are intimately associated. Therefore, the expression of the transcription factor NFkB, which has a pivotal role in the inflammatory response [37], was investigated in AAA and healthy samples. The results perfectly aligned with the expression of the p53 protein. Indeed, a high number of NFkB-positive cells was detected in the tunica adventitia and in the boundary between the tunica intima and media of H samples. NFkB is the main regulator of inflammation, monitoring the expression of inflammatory target genes such as TNF, IL-1 and IL-6, chemokines (MCP1, CCL3, CCL5, CXCL1, and CXCL2), and inflammation-related enzymes (COX2). It has been observed that the suppression of NFkB signaling in AAA mouse models is responsible for the downregulation of IL6 and the strongly reduced infiltration of monocytes in the pathological vascular wall [30]. Our findings support the close relationship between cellular damage and the early steps of inflammation, even in the segments of the vascular wall adjacent to the aneurismatic regions, which suggests that inhibiting the NFkB signaling pathway may be a successful therapeutic approach for reducing the progression of the disease. Despite the lack of inflammatory infiltrate and the morphological integrity of the vascular wall of the healthy regions, the immunohistochemical results demonstrated the presence of the early events of inflammation in H vascular samples, providing evidence of an inflammatory microenvironment in the states preceding aneurysm development. We think that the low number of p53- and NFkB-positive cells in AAA samples is tightly linked to the high level of necrosis observed in the pathological wall.

## 4. Materials and Methods

### 4.1. Sample Collection and Processing for Light Microscopy Analysis

AAA samples were collected in collaboration with the Unit of Vascular Surgery, “Santa Maria delle Croci”, Ravenna Hospital, from 2 patients (1 male and 1 female) during repair surgery while following the guidelines of the Ethics Committee of Ravenna Hospital and in accordance with the Code of Ethics of the World Medical Association. Healthy samples of abdominal aorta consisted of the most external segments of the removed pathological aorta, adjacent to the aneurismatic regions. The tissue donation was based on a “no objection” system for the coded anonymous use of waste tissue left over from surgical procedures. “No objection” negates the need for individual informed consent [38].

Soon after collection, small fragments of the vascular wall were largely washed in phosphate-buffered saline (PBS) and fixed in 4% paraformaldehyde in PBS for 24 h at 4 °C. After several washes in PBS, the samples were dehydrated in ascending graded solutions of ethanol and embedded in paraffin. Paraffin sections of 6 μm were obtained with an automated rotary microtome (Leica Microsystems Srl, Cambridge, UK) and collected on Superfrost glass slides (Carl Roth, Karlshure, Germany).

### 4.2. Histological Staining for the Morphological Analysis of the AAA

Air-dried sections were deparaffinized in xylene, rehydrated through descending graded alcohol solutions to water, and stained with HE, Verhoeff–van Gieson staining, and via Goldner’s Masson trichrome procedure. Regarding the HE protocol, different slides of AAA and healthy vascular walls were stained with an HE kit (Histo-Line Laboratories, Milano, Italy) by following the manufacturer’s instructions. Briefly, the samples were stained with hematoxylin solutions for 10 min at room temperature, washed with running water for 8 min, and then stained with eosin for 1 min at room temperature. Finally, the slides were rapidly dehydrated in ascending ethanol solutions and xylene, and then mounted with DPX mounting medium (Merck KGaA, Darmstadt, Germany).

To evaluate the elastic component in the vascular wall, the Verhoeff–van Gieson staining procedure was carried out. Briefly, after hydration of the slides with water, the samples were stained with Verhoeff’s hematoxylin (5% alcoholic hematoxylin, 10% ferric chloride Lugol’s iodine) for 30 min, followed by a differentiation step with a 2% ferric chloride solution. Then the slides were stained with van Gieson solution (1% acid fuchsin in saturated picric acid) for 5 min. Finally, the slides were dehydrated in ascending ethanol solutions and xylene, and mounted with DPX mounting medium.

To evaluate the presence of fibrotic areas and inflammatory infiltrate, Goldner’s Masson trichrome staining was carried out on all the vascular samples by following the manufacturer’s instructions (Bio-optica, Milan, Italy). Briefly, the slides were dewaxed, washed in distilled water, and stained with Weigert’s iron hematoxylin solution for 10 min at room temperature, followed by 4 min with a picric acid solution. Then the slides were stained with a ponceaus acid fuchsin solution for 4 min and differentiated with a phosphomolybdic solution for 10 min at room temperature. Finally, the samples were stained with a light green solution for 5 min, washed with distilled water, dehydrated rapidly through ascending ethanol solutions and xylene, and mounted with DPX mounting medium. All the slides were observed under a Leica light microscope (Leica Microsystems Srl, Cambridge, UK). The quantitative analysis of the elastic and fibrous areas was assessed by counting the area of five fields for each of three slides per sample at 20× magnification using Image J-2 software (National Institutes of Health, Bethesda, MD, USA). The data were represented as fold increases relative to the healthy control sample.

### 4.3. Transmission Electron Microscopy (TEM)

Small fragments of AAA and healthy abdominal aortic samples were fixed with 2.5% (*v*/*v*) glutaraldehyde in a 0.1 M cacodylate buffer for 24 h, post-fixed with a solution of 1% (*w*/*v*) osmium tetroxide in a 0.1 M cacodylate buffer, and embedded in epoxy resin after a graded-acetone serial dehydration step. The embedded samples were sectioned into ultrathin slices (100 nm thickness), stained with a uranyl acetate solution and lead citrate, and then observed using a CM10 Philips transmission electron microscope (FEI Company, Eindhoven, The Netherlands) at an accelerating voltage of 80 kV. The images were recorded with a Megaview III digital camera (FEI Company, Eindhoven, The Netherlands).

### 4.4. Immunohistochemistry

The paraffin sections were dewaxed and then hydrated in 70% ethanol. Endogenous peroxidase activity was blocked with 3% H_2_O_2_ in 70% ethanol at room temperature for 30 min. After washing them in 70% ethanol, the sections were hydrated in water and washed in PBS at a pH of 7.4 for 5 min. Antigen retrieval was carried out using a solution of 10 mM sodium citrate in PBS for 30 min at 60 °C. Non-specific antibody binding was blocked with 5% bovine serum albumin (BSA) (Merck KGaA, Darmstadt, Germany) for 30 min at RT. The sections were incubated with the primary antibodies for the nuclear factors NF-kB p65 (Ab #8242, Cell Signaling Technology, Beverly, MA, USA), diluted 1:100; p53 (Ab #2527, Cell Signaling Technology, Beverly, MA, USA), diluted 1:100; and p21^cip1/waf1^ (Ab #2947, Cell Signaling Technology, Beverly, MA, USA), diluted 1:100. Both primary antibodies were diluted in blocking solution and the incubation was carried out at 4 °C overnight. To detect the antigen–antibody reaction, a secondary anti-rabbit antibody was used, followed by diaminobenzidine tetrahydrochloride (DAB) as a substrate solution (Histofine immunohistochemical staining kit, Nichirei Biosciences, Tokyo, Japan). Negative controls were performed by omitting the primary antibodies and omitting the primary and secondary antibodies, followed by DAB. All the samples were observed under a light microscope using an Eclipse E800 Nikon (Nikon, Tokyo, Japan). Representative images are shown. The quantitative analysis of the antibody-stained areas was performed by counting the area of five fields for each of three slides per sample at 60× magnification using Leica Qwin 3.0 software (Leica Microsystems Srl, Cambridge, UK), which allowed the antibody-stained area to be selected and measured.

### 4.5. Statistical Analysis

All the statistical analyses were performed using Prism 6 (GraphPad San Diego, CA, USA), applying Student’s *t*-test between each ante-mortem injury and the respective control sample. The differences were considered significant at *p* < 0.05.

## 5. Conclusions

**Figure 8:**
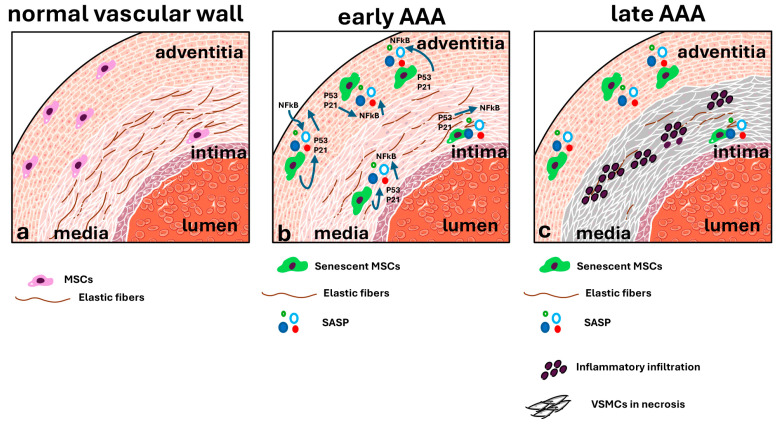
Schematic representation of an AAA’s onset and development based on MSCs’ senescence. (**a**) In a normal vascular wall, a low number of MSCs are localized in the tunica media and adventitia. In physiological conditions, MSCs have a crucial role in vascular remodeling and repair. (**b**) During aging, DDR can occur in MSCs, activating the p53/p21 pathway and inducing a senescent state. Senescent MSCs are resistant to apoptosis and release several inflammatory factors (SASPs) under the inflammatory regulator NFkB. (**c**) Over a long time, the inflammatory microenvironment supports the infiltration of leucocytes in the vascular wall, which actively contribute the matrix proteolysis and elastolysis responsible for the weakness of the vascular wall. In such an inflammatory environment, vascular cells like VSMCs are induced to cell death.

Our results show the presence of a few senescent cells in the vascular wall of the AAA segments, surrounded by highly inflamed segments of the vascular wall adjacent to the pathological areas. These results support the role of senescent cells in supporting inflamed microenvironments by producing SASP factors and creating a condition with a strong impact on disease progression (Figure 8).

We are aware that a significant limitation of this study was the involvement of only two cases for the analysis. Considering the results obtained, we plan to increase the number of cases for further investigation to strongly validate our hypothesis. These data belong to a pilot study based on methods and techniques that, although with a highly precise target location, require considerable time to allow for the collection of results from different samples.

## Data Availability

No new data were created.
